# Cervical Cancer Screening Guidelines in the Postvaccination Era: Review of the Literature

**DOI:** 10.1155/2020/8887672

**Published:** 2020-11-05

**Authors:** Carlo A. Liverani, Jacopo Di Giuseppe, Luca Giannella, Giovanni Delli Carpini, Andrea Ciavattini

**Affiliations:** ^1^Department of Gynecology, Humanitas San Pio X, Milano 20159, Italy; ^2^Woman's Health Sciences Department, Gynecologic Section, Polytechnic University of Marche, Ancona 60100, Italy

## Abstract

Cervical cancer is relatively rare in high-income countries, where organized screening programs are in place, as well as opportunistic ones. As the human papillomavirus (HPV) vaccination rates increase, the prevalence of cervical precancers and cancers is going to decrease rapidly very soon, even if, in the most optimistic scenario, it is unlikely that optimal vaccination coverage will be achieved. Then, the optimal screening paradigm for cervical cancer prevention in the postvaccination era is still debated. Screening guidelines are being developed with the aim of reducing the number of tests a woman needs during her lifetime, in order to receive the maximum benefit from screening, while decreasing potential harms that may result with the use of a screening strategy (overdiagnosis, overtreatment, anxiety, and costs). With this purpose in mind, new management guidelines for cervical cancer screening abnormalities are recommendations based on risks, not on results. This review aims to summarize the process that led to the introduction of the HPV DNA test in screening programs and the different screening strategies. Moreover, it aims to introduce the new risk-based guidelines for the future, where full HPV genotyping can resize the risk on the basis of specific high-risk genotypes. In the same way, the data regarding HPV vaccination could be introduced as soon as women vaccinated with the nonavalent vaccine reach the screening age, with the recommendation of a prolonged screening interval.

## 1. Introduction

Cervical cancer is the seventh most common cancer in European women, accounting for about 3.8% of the total [1]. It is relatively rare in high-income countries, where organized screening programs are in place, as well as opportunistic ones.

Multiple studies have provided mounting evidence supporting human papillomavirus (HPV) DNA-based screening provides greater protection against invasive cervical carcinomas compared with cytological screening [1–4]. Nonetheless, HPV testing has created confusion for many specialists as well as for most of the patients, who often misinterpret the implication of a positive result. When a new test is introduced into daily clinical practice, a misuse is always expected. If the new test is introduced in a screening guideline, then it will be employed even more frequently out of recommendations, because it is presumed to be very effective.

Since HPV DNA testing has been introduced for cervical cancer screening in women aged 30 to 65 years both in the American and in the European guidelines [5–10] and the costs of the new assays have declined and become affordable, HPV tests are being more frequently used in situations where they are not needed. It is well known that the natural history of HPV infections implies frequent clearance of the virus in immunocompetent patients, as well as high rates of spontaneous regression of low-grade lesions [11–13]. However, not rarely clinicians start treating HPV infections detected by molecular methods with surgery, laser, cryotherapy, interferon, and 5-florouracil. Then, multiple preventive, diagnostic, and therapeutic activities are initiated, both in women and in their partners, with strict follow-up programs, more tests, and more interventions. What many health professionals actually do is test women under 25 years of age: rescreen every 1-2 years, test for low-risk HPV types, test anal, vulvar, penile, and oral sites, and test male partners [14].

All these indications are not recommended and may lead to wrong decisions, with well-documented but poorly recognized ill effects [15, 16]. Overtreatment refers not only to the well-studied unfavorable obstetric outcomes following cervical conization procedures [17], but also to possible cervical stenosis or even hematometra requiring surgery, damage to the uterine cervix compromising regular follow-up, and a heavy psychological burden to women and their sexual partners. Intricate algorithms are not easy to follow and poor adherence to guidelines recommendations affect the prerequisite upon which HPV testing strategies are based [18, 19]. Moreover, while HPV primary testing could assume a central role within an organized vaccination and screening program, the opportunistic screening environment in some countries does not yield itself to primary HPV screening and may result in underscreening or lack of screening [20].

Similarly, invasive cervical cancer disproportionately affects women without sufficient access to care, with higher rates among minority groups in higher-income countries and women in low-resource regions of the world [21]. Since equity in access is one of the main objectives of organized screening programs, the introduction of HPV test may suffer from the effect of social inequality: some authors speculated noncompliance to protocols and interaction with opportunistic screening can increase the existing disparities because HPV will be much more expensive than the Pap test. Moreover, longer intervals may disrupt women's habits and thus have detrimental effects on coverage [22].

The same disparity also exists for HPV vaccination: only in industrialized countries, a decline of cervical precancers and cancers is expected, with the increase of the vaccination rate. However, even in the most optimistic scenario, it is unlikely that optimal vaccination coverage will be achieved [23, 24]. Then, what could be the optimal screening paradigm for cervical cancer prevention in the postvaccination era?

This review aims to summarize the process that led to the introduction of the HPV DNA test in screening programs and the different screening strategies in some of the main areas of the world. Moreover, it aims to introduce the new risk-based guidelines for the future, in the postvaccination era.

This review was based on literature search in PubMed (published before October 2020), concerning national and international screening guidelines for cervical cancer.

## 2. Materials and Methods

### 2.1. Accuracy of the Screening Test

All women in the world, regardless of their sexual orientation, must be considered candidates for cervical cancer screening. However, in any population, with any screening program and with any test employed, there will always be some cases of cervical neoplastic and preneoplastic lesions that will not be detected; no test is perfect and the advantages and disadvantages must be carefully weighed in each local reality, when deciding which test or which tests should be adopted.

The sensitivity of conventional cytology, often reported around 50%, was higher than 80% in some studies [25, 26]. In a Japanese study, considering high-grade squamous lesions, the sensitivity of the Pap test in detecting cervical cancer was 94.7% [27]. Overall, the sensitivity of the Pap test varies from 50% to 80%, while the specificity from 70% to 90%.

A large number of studies evaluated molecular biology tests (HPV tests) in primary screening [28, 29]. The sensitivity of the HPV test had often been assessed for cervical intraepithelial neoplasia (CIN) lesions or worse (CIN 2+) [30], but when it was assessed for CIN 3 lesions or worse (CIN 3+), the sensitivity of the HPV test was comparable to that of conventional cytology. In any case, the higher detection of CIN did not lead to an absolute reduction in the incidence of invasive cancers, given the high probability of nonprogression [31]. Both the specificity and the positive predictive values were lower for the HPV test compared to the Pap smear. The combination of the Pap test and HPV test showed greater sensitivity than cytology, but less specificity [32]. In two Italian trials [33, 34], although a CIN 2+ lesion was detected more often with the combination of the Pap test and HPV test compared to cytology alone, the positive predictive value was lower with the combination of the two methods. Regarding HPV testing with cytological triage, a Swedish study showed that the increased incidence of CIN 2 lesions diagnosed at the initial screening was not followed by a statistically significant reduction in CIN 2 lesions to a subsequent screening. Therefore, although the combination of the HPV test and cytology seems to increase the sensitivity, it is known that many lesions can often regress spontaneously [35]. Expanding the detection of CIN could increase overdiagnosis, since the vast majority of low-grade lesions regress in 10 years or less (CIN 1 and 2 regress in 87.7% and 82.9% of the cases, respectively; CIN 1 and 2 progress to a more severe lesion in 9.9% and 32.0% of the cases, respectively) [13]. In a randomised controlled trial (the ARTISTIC study), when combining the first and the second round of screening, the proportion of women with CIN 3+ lesions was similar in the Pap test group and in the Pap test/HPV test group; the sensitivity of thin-layer cytology was slightly higher than that of the HPV test with cytological triage or the Pap test with the HPV triage test also in detecting CIN 3+ lesions [36].

Eventually, sensitivity should not be overstated [37]. As reported above, less than a third of high-grade lesions progress to cancer [13, 38, 39]. Unfortunately in the United States, one doctor out of four is testing for both high-risk and low-risk HPV types; about 60% of health care providers choose cotesting (the Pap test and HPV test) under the age of 30 years; there is a widespread use of molecular tests for high-grade lesions already histologically confirmed; the cotest is repeated annually or every two years. All these habits are wrong and may lead to waste of resources and possible harms caused by overtreatment [40–42]. Moreover, according to the data reported by the World Health Organization (WHO), there are several HPV testing products available (29 tests) [43]. Some of these are able to detect a group of high or low-risk genotypes. Only a small part is able to type the viral genotype, in particular for HPV 16 and 18. All these make interpretation of the result even more difficult. In Italy, there are even 12 different types of tests validated and usable for screening, but which provide different results [44]. Another concern raised is that some studies have demonstrated that up to 10% of cervical tissues in US tumor registries do not contain detectible HPV [45–48] and that 37% of cervical adenocarcinomas around the world are HPV negative [49].

Nonetheless, the publication of Ronco et al. in 2014, analyzing the follow-up for the four main randomised controlled trials on the effectiveness of screening based on the HPV test, definitely showed that the cumulative probability of developing cervical cancer in women included in the Pap test program was greater than those included in the Pap test/HPV test program, leading to the conclusion that HPV-based strategies confer 60–70% greater protection than cervical cytology [2]. This study was not free from criticism [50], but influenced many associations to change their current guidelines.

## 3. Cervical Cancer Screening around the World

Table 1 shows the worldwide HPV vaccine coverage among female population aged 10–20 [51], the cervical cancer incidence (by continent) [52], and the health care systems (by countries).

-  Nonuniversal insurance system: Bangladesh, Egypt, Ethiopia, Jordan, Kenya, Nigeria, Paraguay, Tanzania, Uganda, the United States, and Yemen
-  Universal government-funded: Australia, Bahrain, Bhutan, Botswana, Brazil, Brunei Canada, Cuba, Denmark, Finland, Georgia, Greece, Iceland, India, Ireland, Italy, Kuwait, Malaysia, Malta, New Zealand, North Korea, Norway, Oman, Portugal, San Marino, Saudi Arabia, South Africa, Spain, Sri Lanka, Sweden, Taiwan, Trinidad and Tobago, and the United Kingdom
- Universal private health insurance system: Israel, Liechtenstein, Netherlands, and Switzerland
- Universal public insurance system: Albania, Andorra, Belgium, Bulgaria, China and Hong Kong, Colombia, Croatia, Czech Republic, Estonia, France, Hungary, Iran, Japan, Latvia, Lithuania, Luxembourg, Monaco, Moldova, Montenegro, Poland, Qatar, Romania, Russia Serbia, Singapore, Slovakia, Slovenia, South Korea, Ukraine, and the United Arab Emirates
- Universal public-private insurance system: Algeria, Argentina, Austria, Chile, Cyprus, Germany, Mexico, Peru, and Turkey

### 3.1. Europe

In Europe, about 60,000 new cases of cervical cancer are being diagnosed every year, with 25,000 deaths per year [53]. Despite the implementation of vaccination programs, screening for cervical cancer precursors and treatment of detected lesions will remain the most effective tool for cervical cancer prevention in the medium-long term. Cervical cytology has been the cornerstone of European screening programs: according to data from the International Agency for Research on Cancer, Pap smears performed every 3–5 years reduce the incidence of cervical cancer by 80% [54]. Screening is “organized” in the Nordic countries, in the United Kingdom, in Holland, in Poland, Croatia and Slovenia, Belgium, and the Czech Republic, and in many parts of Italy [54, 55]; screening is “opportunistic” in the remaining countries (with a low cost-effectiveness ratio: overscreened women and underscreened women).  Table 2 shows the programmes of cervical cancer screening and human papillomavirus (HPV) vaccination in European countries [55].

The Council of the European Union (which represents the 27 member states) has always recommended the cervical cancer screening intervals reported in Table 3 [56].

A supplement to the second edition of the European guidelines [6] was published in September 2015 introducing the HPV DNA test alone, as the primary screening test in European countries (as opposed to the American guidelines, which recommended cotesting), together with the implementation of HPV vaccination programs. Notably, it was reported that

1. the primary test for the detection of high-risk oncogenic HPV types can be used within an organized screening program,
2. HPV test is not recommended outside of organized programs. Cotesting (the Pap test and HPV test) should be avoided: only one primary test, Pap test or HPV test, should be employed,
3. routine screening with HPV tests should be used starting at the age of 35 and in any case not below 30 years (in the age group between 30 and 34 years, the available evidence was not considered sufficient to recommend HPV DNA testing). Under 30–34 years of age, the recommended screening test is the Pap test,
4. the screening interval in women with the HPV negative test must be at least 5 years, with the possibility of extension up to 10 years, depending on the age and history of the patient. Screening can cease at the age of 60–65, provided that the woman has a recent negative test, and
5. the management of a woman with a positive HPV test involves triage with cervical cytology, while direct referral to colposcopy is not recommended.

Table 4 reports cervical cancer screening intervals with the HPV test and Pap test, according to European Union recommendations.

Furthermore, the European Council recommends high quality standards in all phases of the program (invitation, screening, diagnosis, treatment of detected lesions, and post-treatment follow-up), and urges to discourage opportunistic screening as well as to refrain from introducing new primary screening programs in the absence of population studies [6, 56].

Despite the consistent evidence that organized programs are more efficient than opportunistic screening, and despite the recommendations of the European Council, the health authorities of many European countries have not yet implemented a nationally organized cervical cancer screening program [57, 58]. The Italian health system, by means of all the regions in the country, is committed to changing the screening program by the end of 2020. However, a great variability still exists from region to region [59], with local realities employing cervical cytology every 3 years from 25 to 64 years of age, and regions employing cytology from 25 to 29 years and then shifting to HPV testing every 5 years from 30 to 64, and situations in which screening is spontaneous and unorganized, with differences even within a same region. On the other hand, in Holland, the HPV DNA test program was established at a national level, at 5-year intervals from 30 to 40 years, and at 10-year intervals from 40 to 60 years. No screening is recommended for women under 30 and over 64 years [60].

In the United Kingdom, cervical screening follows the European Guidelines, but liquid-based cytology is recommended instead of conventional cytology (Table 5). HPV screening is being piloted across England to assess how this approach can be used across the programme as a whole [61, 62].

### 3.2. The Russian Federation

Cervical cancer is the second cause of death in women in Russia. About 70% of all cancers are still diagnosed in advanced stages. No organized screening program is in place, but the Minister of Health recommends screening as for the WHO and European standards [63].

### 3.3. The United States of America

The American guidelines are the most comprehensive, undoubtedly validated by sufficient scientific evidence in their effort to achieve the maximum cost-effectiveness ratio, but certainly complex in practical management. Recommendations have a “grading” with 5 degrees (“A” when the benefit is high, “B” when it is moderate, “C” when it is low, “D” when the disadvantages outweigh the advantages, and “I” when the scientific evidence is not yet sufficient to determine the balance between benefits and harms), and quality of evidence at 3 levels (“high,” “moderate,” and “low”). The recommendations were shared, until July 2020, by the American Cancer Society (ACS), the American Society for Colposcopy and Cervical Pathology (ASCCP), and the American Society for Clinical Pathology (ASCP) (Table 6) [7, 8]. In July 2020, the ACS produced a guideline update that leads to substantial changes [10]. Until July 2020, the ACS was in accordance with other American societies.

The most recent ACS guideline (July 2020) [10] calls for substantial changes to the screening method: the ACS recommends that individuals with a cervix initiate cervical cancer screening at age 25 y and undergo primary HPV testing every 5 y through age 65 y (preferred). If primary HPV testing is not available, individuals aged 25–65 y should be screened with cotesting (HPV testing in combination with cytology) every 5 y or cytology alone every 3 y (acceptable) (strong recommendation). A cotesting or cytology testing alone are included as acceptable options for cervical cancer screening because access to primary HPV testing with a test approved by the FDA for primary screening may be limited in some settings. The guideline is transitional; that is, options for screening with cotesting or cytology alone are provided but should be phased out once full access to primary HPV testing for cervical cancer screening is available without barriers [10].

The U.S. Preventive Services Task Force (USPSTF), probably the most rigorous in the formulation of the guidelines, prepared a separate document in which women aged 30 to 65 years should be screened every 3 years with cervical cytology alone, or every 5 years with high-risk HPV testing alone or in combination with cytology [11].

The cotest (the Pap test + HPV test) is only offered in women aged 30 to 65 years, provided that the 5-year rescreening interval is respected. This approach is defined “preferable” for the ASCCP/ASCP, while it is defined “acceptable” for the ACS/USPSTF. HPV testing as a standalone test (i.e., without cytology) was not recommended.

A study [64] was conducted to verify the ideal strategy for cervical cancer screening, which should offer the maximum sensitivity to minimize the risk of missing the disease, together with the maximum specificity to reduce the rate of false-positive cases. This analysis involved 34,254 women aged ≥30 years, extrapolated from the ATHENA (Addressing the Need for Advanced HPV Diagnostics) trial, with an average age of 44.7 years. 8.4% of women were HPV positive, and 5.7% had an abnormal Pap smear. Ten different screening strategies were compared. The most attractive for the “benefits” versus “harms” analysis was the four strategies that involved the use of the HPV test with genotyping for HPV 16/18 (two of which in the cotest with cytology). The two strategies that would seem to optimize the balance between sensitivity and specificity are the cotest with genotyping and triage with Pap LSIL (strategy no. 5) and the HPV test with genotyping and triage with Pap ASC-US (strategy no. 9). The latter led to a 50% reduction in the number of screening tests required, as well as being slightly more sensitive and requiring slightly less colposcopies to detect a CIN 3 or worse lesion. In conclusion [64], according to the authors, to employ in primary screening, a strategy involving the use of the HPV test with triage of HPV-positive women through a combination of genotyping for HPV 16/18 and cytology would provide a good balance between obtaining of maximum sensitivity and specificity, limiting the number of colposcopy referrals. However, it must be noted that the study in question is a post hoc analysis (the ATHENA study had another objective); moreover, it was impossible to know how many high-grade lesions or worse would have been diagnosed at the next screening or during the follow-up.

Last but not least, compliance with recommendations to follow-up is essential; otherwise, the assumptions of the entire algorithm collapse. These strategies are therefore a little too intricated, difficult to apply in real practice as far as regards doctors' attitudes as well as patients' preferences. The American algorithms are also very complex for professionals and, in their attempt to maintain a correct cost-effectiveness ratio, they risk being difficult to use and above all not being respected [7–9, 18, 19, 65]. For instance, the most common rescreening interval after a negative HPV result was found to be only 12 months in the United States [60]. Interim Clinical Guidance for use of primary high-risk human papillomavirus testing for cervical cancer screening has also been published introducing in the USA the use of the HPV test as the only primary screening method [3, 66]. However, these works are burdened by heavy conflicts of interest (Merck, GSK, Hologic, Roche, Gen Probe, Becton-Dickinson, Cepheid, and Genentech). In Wright's article, even one of the sponsors (Roche Molecular Systems, Pleasanton, CA) was involved in all aspects of the design and conduct of the study: collection, management, analysis, and interpretation of the data; two employees of the pharmaceutical company were integral to the preparation of the manuscript, and the sponsor reviewed the final version of the manuscript [3].

In a conflict-free article [67], however, the Cobas test was evaluated on more than 33,000 Pap smears, detecting a 59.7% negative test rate in cases with abnormal cytology and a 38.2% negative test rate in a subset of histologically confirmed CIN 2–3 lesions. Obviously, the authors' conclusion is that the HPV Cobas test cannot be used in primary screening, as it would provide a false guarantee to doctors and patients, delaying appropriate treatment.

The latest recommendations from the Atlanta Centers for Disease Control and Prevention (CDC) (June 2015) clearly stated that the HPV test should not be recommended for screening for cervical cancer as a standalone test (i.e., without a concurrent Pap test) [68].

Finally, in 2020, the ASCCP has introduced new management guidelines for cervical cancer screening abnormalities, revolutionizing the future of risk-driven recommendations for this topic (see below) [69].

### 3.4. Canada

In 2011, around 1.300 new cases of cervical cancer were recorded in Canada (with about 350 deaths). There has been no reduction in cervical cancer mortality in Canada since 1970 in women aged 20–24 years (i.e., since the start of screening) [70].

The recommendations follow a simpler “grading” than the USA, with only 2 degrees: “strong” or “weak”; on the other side, the quality of evidence is divided into 4 levels: “high,” “moderate,” “low,” and “very low.” Three-year intervals are the right balance between the small potential to increase the benefits obtainable with shorter intervals and the greater potential for harms resulting from the increase in tests and procedures related to more frequent screening. Screening by the HPV test, alone or in combination with the Pap test, is not recommended (as there was insufficient evidence on its effect on mortality and incidence of invasive cancer). Canadian guidelines also state that organized screening is more effective than opportunistic screening. The sensitivity and specificity of thin-layer and conventional cytology are similar. An increase or decrease in screening may be appropriate for women with different risk profiles. The recommendations are reported in Table 7.

These recommendations do not apply to women with cervical cancer symptoms or previous abnormal screening test results; women whose uterine cervix has been completely removed; immunosuppressed women for HIV, organ transplants, chemotherapy, or chronic use of corticosteroids; and women with reduced life expectancy so that they cannot benefit from screening. Finally, it is recommended that clinicians be aware of the values, preferences, and beliefs of each woman. HPV DNA testing as primary screening strategy is being introduced in many provinces.

### 3.5. Latin America

Screening programs for cervical cancer in Latin America and the Caribbean had not been successful in many situations, and incidence and mortality are still high. Even after the introduction of screening programs, incidence and mortality for cervical cancer did not change in many countries, because the coverage of screening is inadequate in rural areas [71, 72]. Of note, Chile, Colombia, Mexico, Peru, and more recently Argentina, are actively evaluating and proposing new strategies for cervical cancer control [73]. In consequence, countries such as Chile, Colombia, and Costa Rica are registering reductions in mortality rates for cervical cancer [72].

### 3.6. Australia

Until 2017, Australian recommendations were very simple and effective. The first Pap smear was proposed between 18 and 20 years of age, or 1–2 years after the onset of sexual activity (depending on which of the two events occurs later) and then repeated every 2 years until 69 years.

The “National Cervical Screening Program” was revised in 2017 [74]. Therefore, starting from this date, HPV testing was introduced for primary screening (Table 8).

The new cervical cancer screening pathway is a risk-based approach in which partial genotyping is used to classify the type of HPV into one of two groups: oncogenic HPV 16/18 or oncogenic HPV types not 16/18, as a pooled result. The Australian risk-based approach, according to HPV test result, is reported in Table 9.

Cotesting is recommended for symptomatic patients (usually abnormal vaginal bleeding) requiring investigation, patients exposed to diethylstilbestrol (DES), patients undergoing Test of Cure (TOC) surveillance, and patients who have been treated for glandular abnormalities.

### 3.7. Japan

Cervical cancer is the ninth leading cause of cancer death in women in Japan. In 2018, about 13.277 new cervical cancer cases were diagnosed in Japan. Recommendations have a 5-degree “grading” as in the USA (“A” and “B” grades indicate when recommendations are valid for both mass and opportunistic screening programs, “C” when they are not valid for mass screening, “D” when they are neither valid for mass nor opportunistic screening, and “I” refers to screening methods in which there is insufficient evidence), and quality of evidence up to 8 levels (based on the type of study: design, quality, and consistency of the study) [75]. Screening guidelines were formulated on the basis of the balance between benefits and harms recommendations for population-based and opportunistic. Five cervical cancer screening methods were evaluated (three of them with the HPV test), after considering 3.450 articles published from January 1985 to October 2007. After a systematic review of the literature, 161 articles were selected and 66 were confirmed. The results of 33 studies were considered satisfactory and the scientific evidence sufficient to evaluate the effect of screening by conventional cytology. The accuracy of thin-layer cytology was equivalent to that of conventional cytology. Although HPV tests and combination methods have shown high sensitivity, no studies have evaluated the reduction in mortality from cervical cancer. In mass screening, as well as in opportunistic screening, conventional or thin-layer cytology is recommended, given sufficient scientific evidence (Grade B). Cervical cancer screening using the HPV test alone or in combination with cervical cytology was not recommended in mass screening, given insufficient scientific evidence (Grade I). Since 2003, screening in Japan has been carried out, as reported in Table 10.

Given the different health system in Japan compared to other countries, it was not considered appropriate to modify the current recommendations. To reduce cervical cancer mortality in Japan, it is rather necessary to improve adherence to screening programs and the appropriateness of managing the lesions detected [75]. At the same time, a retrospective study on 3.804 women aged ≥20 years followed for 5 years (from 2005 to 2010) [76], assessed how the incidence rates of cytological abnormalities, CIN, and cervical cancer vary according to age. Under the age of 40, about 5% of women had an abnormal Pap test result, about 3% had CIN, and 0.5% developed a CIN within two years. In women aged between 40 and 49, less than 4% had a cytological abnormality within two years and approximately 5% within three years. In the group between 50 and 59 years old, less than 2% had a cytological abnormality within two years and about 3% within three years. While 0.1% of women developed a CIN in the 40–49 year group within three years, none in the 50–59 year group developed a CIN within three years. In women aged 60 and over, less than 3% had an abnormal Pap test result, less than 0.5% developed a CIN within 5 years, and none developed cancer in the same period. According to the results of this study, the author proposed the following modification [76]: Pap test every 2 years between 20 and 40 years, every 2 or 3 years between 40 and 59 years, and every 5 years after 60 years.

### 3.8. China

Although in China today there are several excellent programs for cervical cancer screening in large cities, these programs cover only a minimal part of the whole population in the country. To achieve a significant reduction of the incidence of invasive cervical cancer with concomitant reduction in the death rates, a population-based screening program reaching all women at risk should be instituted. Since it would take almost two decades to build up the infrastructure for a conventional approach, it has been decided to employ newly developed technology, which could be implemented in a couple of years [77].

### 3.9. Cervical Cancer Screening in Developing Countries

In Sub-Saharan Africa (SSA), although incidence rates vary from country to country, cervical cancer is the second most common cancer of reproductive-aged women [78]. In 2004, the Malawian Ministry of Health has introduced a comprehensive cervical cancer screening programme using visual inspection with acetic acid (VIA) and cryotherapy [79]; objective of the screening programme was to screen 80% of Malawi's eligible women. Despite the programme has not achieved its goal, the percentage of women screened increased from 9.3% to 26.5%, but with major problems related to “lost to follow-up” of positive patients. In Nigeria, the most populous country in Africa, almost 15,000 new cases of cervical cancer are diagnosed annually [80]. Nigeria has developed a National Cancer Control Policy that incorporates implementation of HPV DNA testing/VIA with the treatment of precervical lesions and implementation of HPV vaccination programmes [80]. Currently, HPV vaccination is available on a private basis only, but efforts are ongoing to incorporate HPV vaccination into their routine immunization programmes. In 2013, it became the first African country to implement a national prevention programme for cervical cancer [81]. The Rwanda national HPV vaccination model could be the first step towards cervical cancer elimination in Africa [80].

In the Middle East and North Africa, the first steps to implement national screening programmes based on visual inspection tests are being currently complete [82]. The cervical cancer screening coverage in Southern Africa ranges between 4.1 and 38.0% [83]. One preventive strategy used in South Africa was the introduction of the HPV vaccine. In 2014, a national school-based program for the HPV bivalent vaccine was introduced in all public schools, targeting girls in Grade 4 (aged ≥9 years old) with a two-dose (6 months apart) schedule [84]. In India, guidelines for population-based screening programmes for cervical cancer are available [85] and are based, largely, on visual inspection tests; however, screening coverage is still very low.

Few population-based cervical cancer screening programs had been implemented in Western Asian and Middle Eastern Arab countries, with low awareness about cervical screening among Arab Muslim. Factors affecting the coverage of cervical cancer screening practices were the absence of organized screening programs, low screening knowledge among women, healthcare professionals' attitudes toward screening, pain and embarrassment, stigma, and sociocultural beliefs [86].

## 4. The New Risk-Based Guidelines for Cervical Cancer Screening Abnormalities, in the Postvaccination Era

Guidelines are being developed with the aim of reducing the number of tests a woman needs during her lifetime, in order to receive the maximum benefit from screening, while decreasing potential harms that may result with the use of a screening strategy (overdiagnosis, overtreatment, anxiety, and costs). With this purpose in mind, the ASCCP has recently introduced new management guidelines for cervical cancer screening abnormalities, where recommendations are based on risks, not results [69, 87–89].

The American guidelines for the management of the altered screening test are an evolution also with respect to the innovative Australian guidelines [74].

This change from primarily test results to primarily risk-driven guidelines was made possible through careful evaluation of specific clinical action thresholds. HPV DNA testing is the basis for risk estimation, with reflex cytology as a triage test for all HPV-positive results. Surveillance thresholds work on the principle of similar management for similar risks. Thus, a set of probabilistic risk models were studied within a minimum amount of data available, to provide a personalized estimate of risk for the high-grade cervical disease over time. Depending on age (<25 years and ≥25 years), current test results (cytology, HPV testing or both), and previous screening history when available, five different scenarios could be identified [66]:

1. Return to routine screening (preferably HPV-based), when the 5-year risk of a high-grade cervical lesion is less than 0.15%
2. Surveillance with repeat testing in 3 years without colposcopy, when the 5-year risk ranges from 0.15% to 0.54%
3. Surveillance with repeat testing in 1 year without colposcopy, when the 5-year risk ranges from 0.55% to 3.9%
4. Colposcopy, when there is an immediate risk of high-grade disease ranging between 4% and 24%
5. Treatment, when the immediate risk of high-grade disease is ≥ 25% (with the option of expedited treatment, i.e., not preceded by a confirmation biopsy, when the risk is ≥ 60%)

This shift represents a great leap forward to precision medicine, where risks are individualized and care is person-centered.

The risk of progression to severe high-grade CIN and cancer is strongly associated with HPV genotype and genotype-specific persistence. Different oncogenic genotypes entail different risks, with HPV 16 ranking the highest, and HPV 66 the lowest [90]. Extended genotyping (i.e., assays reporting all 14 high-risk genotypes) enables personalized clinical management for women screened through the primary HPV paradigm. By stratifying genotype-specific results, it is possible to assign women to different interventions. Highest risk genotypes (HPV 16, 18, 31, and 33) together with moderate risk genotypes (HPV 45, 52, and 58) should be managed differently than the types at lower risk (HPV 35, 39, 51, 56, 59, 66, and 68), which could be followed up less stringently. Since guidelines are intentionally built to allow updates to incorporate new tests (e.g., full HPV genotyping or dual staining with p16/Ki-67 immunocytology), as well as to adjust for decreasing incidence of high-grade lesions following HPV vaccination [66], it can be foreseen a prolonged rescreening interval among the vaccinated population. Quadrivalent HPV vaccine is available since 2006, while nonavalent HPV vaccine since 2016. Women vaccinated with the latter are to be considered protected right against the seven abovementioned HPV genotypes at higher risk. The information obtained can be easily introduced into the data required to retailor risk estimates, though algorithms would certainly result even more intricate. To overcome the challenge, national societies are planning to implement algorithms on mobile phones. Apps are already available to assist with the navigation of previous guidelines [91] and a new one has been recently developed by ASCCP. These apps could be upgraded in real time, as new mosaic tiles will become available once validated by clinical trials. In a world where telemedicine and telemonitoring are at hand [92], not only as a support in underserved communities but also when there is a pandemic or other types of crisis, the opportunity to dispose of tech tools to guide health providers through all steps of a complex screening algorithm will be much appreciated.

In conclusion, full genotyping can resize the risk on the basis of specific high-risk genotypes. In the same way, the data regarding HPV vaccination could be introduced as soon as women vaccinated with the nonavalent vaccine reach the screening age, with the recommendation of a prolonged screening interval. Lifetime testing could be cut in half among the vaccinated population, reducing costs, anxiety, and possible overtreatments. Nevertheless, clinicians should always bear in mind that screening focuses on people with no symptoms and that in the presence of risk factors (postcoital bleeding, cigarette smoking, immunosuppression, and unexplained vaginal discharge), women should follow a different pathway, and further investigation may be requested regardless of test results.

## Figures and Tables

**Table 1:** Worldwide human papilloma virus coverages among female population aged 10–20 years, the cervical cancer incidence (by continent), and the health care systems (by countries).

Geographical region	HPV vaccine coverage	Cervical cancer incidence (cumulative risk 0–74) (%)	
Africa	1.2% (0.6–2.0)	Eastern Africa	4.34
		Middle Africa	3.02
		Northern Africa	0.82
		Southern Africa	4.20
		Western Africa	3.47

Asia	1.2% (0.7–1.8)	Eastern Asia	1.09
		South-Eastern Asia	1.86
		South-Central Asia	1.41
		Western Asia	0.45

Europe	36.4% (30.3–42.9)	Central and Eastern Europe	1.58
		Western Europe	0.66
		Southern Europe	0.77
		Northern Europe	0.85

Latin America and Caribbean	22.1% (12.7–32.6)	Caribbean	1.54
		Central America	1.32
		South America	1.56

North America	53.4% (27.1–85.6)	North America	0.62

Oceania	41.1% (21.5–64.0)	Australia and New Zealand	0.55
		Melanesia	2.60
		Polynesia	1.16
		Micronesia	2.03

Data in parentheses are 95% confidence interval.

**Table 2:** Programmes of cervical cancer screening and HPV vaccination in European countries.

Countries	Cervical screening program (starting year)	Screening age	Screening interval	Primary test used	HPV vaccine (national vaccination program) (year of initiation)
Austria	Opportunistic	18+ or 2 years after sexual onset	1	CC	2014

Belgium	Organized population-based, in some regions, rollout ongoing, 2013	25–64	3	CC & LBC	2010

Bulgaria	Opportunistic	NA	NA	NA	-

Croatia	Organized population-based, rollout ongoing, 2012	25–64	3	CC	2016

Cyprus	Opportunistic	NA	NA	N/A	2016

Czech republic	Organized population-based, rollout ongoing, 2008	15+	1	CC	2012

Denmark	Organized population-based, 2006	23–64	3 (ages: 23–59); 5 (ages: 60–64)	LBC (ages: 23–59); HPV primary test (ages:60–64)	2009

Estonia	Organized population-based, 2006	30–59	5	CC	2018

Finland	Organized population-based (depending on the region), 1963	25–65	5	CC, HPV primary testing in some regions	2013

France	Transitioning to organized population-based planned, 1991	25–64	3 (CC), 5 (HPV test)	CC & LBC (ages: 25–64); HPV primary testing in regional pilot projects (ages: 30–64)	2007

Germany	Transitioning to organized population-based planned, 1971	20+	1	CC, HPV primary testing in implementation	2007

Greece	Opportunistic	Sexual onset	1	CC	2008

Hungary	Organized population-based, rollout ongoing, 2003	25–65	3	CC	2014

Ireland	Organized population-based, rollout ongoing, 2008	25–60	3 (ages: 25–44); 5 (ages: 45–60)	LBC	2010

Italy	Organized population-based, rollout ongoing, depending on the region, 1989	25–64	3 (ages: 25–30/35); 5 (ages: 30/35–64)	CC & LBC (ages: 25–30/35); HPV primary testing in some regions (ages: 30/34–64)	2008

Latvia	Organized population-based, 2009	25–69	3	CC	2010

Lithuania	Organized population-based, rollout ongoing, 2004	25–59	3	CC	2016

Luxembourg	Opportunistic	15+	1	LBC	2008

Malta	Organized population-based, piloting, 2015	25–35	3	CC	2012

Netherlands	Organized population-based, 1970	30–64	5	HPV primary testing	2010

Norway	Organized population-based, 2006	25–69	3	CC & LBC, regional pilot for HPV primary testing,	2009

Poland	Organized population-based, 2006	25–59	3	CC (ages: 25–59); regional pilot for cotesting (ages: 30–59)	—

Portugal	Organized population-based, in some regions, rollout ongoing, depending on the region, 1990	25–59	3	Cotesting in some regions	2008
Romania	Organized population-based, in some regions, rollout ongoing, 2012	25–64	5	Cotesting in some regions	—

Slovakia	Transitioning to organized population-based, 2008	23–64	Yearly x 2; then 3 yearly	CC	—

Slovenia	Organized population-based, (2003)	20–64	Yearly x 2; then 3 yearly	CC	2009

Spain	Opportunistic (depending on the region)	25–65	3	CC	2007

Sweden	Organized population-based, 1967	23–60	3 (ages: 23–50); 5 (ages: 51–60)	HPV test replacing CC & LBC	2012

Switzerland	Opportunistic	21/70	2 (ages: sexual onset/21–29); 3 (ages: 30–70)	CC & LBC	2008

The United Kingdom	Organized population-based, 1988	25–64	3 (ages: 25–49); 5 (ages: 50–64)	HPV primary testing in implementation	2008

CC = conventional cytology; LBC = liquid-based cytology; N/A: not available.

**Table 3:** Cervical cancer screening intervals with the Pap test according to the European Union recommendation (depending on the available resources in each member state).

Age	Method and intervals
Onset of screening: between 20-25 and 30 years	Pap test every 3–5 years
End of screening: 60–65 years	

**Table 4:** Cervical cancer screening intervals with the HPV test and pap test, according to the European union recommendation (depending on the available resources in each member state).

Age	Method and intervals
Screening starts between 20-25 and 30 years	Pap test every 3–5 years
An alternative starting at 30–35 years	HPV DNA test every 5–10 years

End of screening: 60–65 years	

**Table 5:** Cervical cancer screening intervals with the Pap test, according to the United Kingdom recommendation.

Age	Method and intervals
<25 years	No screening
25–49 years	Liquid-based cytology every 3 years
50–64 years	Liquid-based cytology every 5 years
≥65 years	Screening for women who have had recent abnormal tests or who have not had an adequate screening test reported since age 50

**Table 6:** Cervical cancer screening intervals with the HPV test and Pap test, according to the American Society for Colposcopy and Cervical Pathology (ASCCP) and the American Society for Clinical Pathology (ASCP).

Age	Methods
<21 years (Grade D)	No screening (in the case of a Pap smear ASC-US: HPV testing is not recommended)

21–29 years (Grade A)	Pap smear every 3 years

30–65 years for women who want to extend their screening interval (Grade A)	Cotest (the Pap test + HPV DNA test) every 5 years

30–65 years in women who choose to be tested more often (Grade A)	Pap smear every 3 years

>65 years (Grade D)	No screening in women with previous adequate screening (i.e., at least two negative results in the last 10 years, with at least one in the last 5) in women with a history of CIN 2 or worse lesions: Continue routine screening for at least 20 years

Screening posthysterectomy (Grade D)	No screening (women with removal of the cervix and no history of a high-grade precancerous lesion in the last 20 years, or cervical cancer ever)

After HPV vaccination (Grade C)	Age-based recommendations for the general population (at the moment according to the same guidelines as women who have not been vaccinated)

**Table 7:** Cervical cancer screening intervals according to the Canadian task force on preventive health care recommendations on screening for cervical cancer.

Age	Method	Grade of recommendation/quality of evidence
<20 years	No screening	Strong/high

20–24 years	No screening	Weak/moderate

25–29 years	Pap smear every 3 years	Weak/moderate

30–69 years	Pap smear every 3 years	Strong/high

>70 a years	No screening if at least three consecutive negative Pap tests in the past 10 years; otherwise, continue screening until three consecutive negative results	Weak/low

**Table 8:** Cervical cancer screening intervals according to the Australian guidelines.

Age	Method and intervals
25–74 years	HPV DNA every 5 years
>74 years	No screening (at the discretion of the caregiver, if the previous results are normal)

**Table 9:** Cervical screening test results for clinician-collected sample according to the Australian guidelines.

Risk of significant cervical abnormalities	HPV test result	Reflex liquid-based cytology result	Recommended management
Low-risk result	HPV not detected	—	Return to screening in 5 years

Intermediate-risk result	HPV not 16/18 detected	Negative, possible LSIL or LSIL	Repeat HPV test in 12 months

Higher-risk result	HPV not 16/18 detected	Possible HSIL or HSIL	Refer to specialist (colposcopy)
	HPV 16/18 detected	Any LBC result	Refer to specialist (colposcopy)

—	Unsatisfactory HPV test	—	Collect new sample for the HPV test in 6–12 weeks

—	HPV not 16/18 detected	Unsatisfactory	Collect new sample for LBC only in 6–12 weeks

**Table 10:** Cervical cancer screening intervals according to the Japanese guidelines.

Age	Method and intervals
>20 years	Pap test every 2 years

## Data Availability

This review was based on literature search in PubMed (published before September 2020), concerning national and international screening guidelines for cervical cancer.

## References

[1] WHO regional office for Europe, available on https://www.euro.who.int/en/health-topics/noncommunicable-diseases/cancer/news/news/2012/2/early-detection-of-common-cancers/cervical-cancer#:%7E:text=Cancer%20of%20the%20cervix%20uteri,programmes%20have%20a%20long%20history

[2] Ronco G., Dillner J., Elfström K. M. (2014). Efficacy of HPV-based screening for prevention of invasive cervical cancer: follow-up of four European randomised controlled trials. *The Lancet*.

[3] Wright T. C., Stoler M. H., Behrens C. M., Sharma A., Zhang G., Wright T. L. (2015). Primary cervical cancer screening with human papillomavirus: end of study results from the ATHENA study using HPV as the first-line screening test. *Gynecologic Oncology*.

[4] Lew J.-B., Simms K. T., Smith M. A. (2017). Primary HPV testing versus cytology-based cervical screening in women in Australia vaccinated for HPV and unvaccinated: effectiveness and economic assessment for the national cervical screening program. *The Lancet. Public Health*.

[5] Tota J. E., Bentley J., Blake J. (2017). Introduction of molecular HPV testing as the primary technology in cervical cancer screening: acting on evidence to change the current paradigm. *Preventive Medicine*.

[6] von Karsa L., Arbyn A., De Vuyst H., Anttila A., Arbyn A., De Vuyst H. (2015). Executive summary. *European Guidelines for Quality Assurance in Cervical Cancer Screening*.

[7] Saslow D., Solomon D., Lawson H. W. (2012). American cancer society, American society for colposcopy and cervical pathology, and American society for clinical pathology screening guidelines for the prevention and early detection of cervical cancer. *CA: A Cancer Journal for Clinicians*.

[8] Massad L. S., Einstein M. H., Huh W. K. (2013). 2012 updated consensus guidelines for the management of abnormal cervical cancer screening tests and cancer precursors. *Obstetrics & Gynecology*.

[9] Moyer V. A., U.S. Preventive Services Task Force (2012). Screening for cervical cancer: U.S. preventive services task force recommendation statement. *Annals of Internal Medicine*.

[10] Fontham E. T. H., Wolf A. M. D., Church T. R. (2020). Cervical cancer screening for individuals at average risk: 2020 guideline update from the American cancer society. *CA: A Cancer Journal for Clinicians*.

[11] US Preventive Services Task Force, Guerra S. J., Curry S. J., Krist A. H (2018). Screening for cervical cancer: U S preventive services task force recommendation statement. *JAMA*.

[12] Winer R. L., Feng Q., Hughes J. P., O’Reilly S., Kiviat N. B., Koutsky L. A. (2008). Risk of female human papillomavirus acquisition associated with first male sex partner. *The Journal of Infectious Diseases*.

[13] Holowaty P., Miller A. B., Rohan T., To T. (1999). Natural history of dysplasia of the uterine cervix. *JNCI Journal of the National Cancer Institute*.

[14] Liverani C. A. (2013). The four steps in the prevention of human papillomavirus-associated neoplasia. *Archives of Gynecology and Obstetrics*.

[15] Liverani C. A., Vercellini P., Frattaruolo M. P., Bolis G. (2014). What is and what should never be: use and misuse of HPV testing in cervical cancer prevention strategies. *Current Women s Health Reviews*.

[16] Solomon D., Papillo J. L., Davey D. D., Cytopathology Education and Technology Consortium (2009). Statement on HPV DNA test utilization. *Journal of Lower Genital Tract Disease*.

[17] Davey D. D., Goulart R., Nayar R., Cytopathology Education and Technology Consortium (CETC) (2014). Statement on human papillomavirus DNA test utilization. *American Journal of Clinical Pathology*.

[18] Kyrgiou M., Athanasiou A., Kalliala I. E. J (2017). Obstetric outcomes after conservative treatment for cervical intraepithelial lesions and early invasive disease. *The Cochrane Database of Systematic Reviews*.

[19] Castle P. E. (2010). Screening: HPV testing for cervical cancer: the good, the bad, and the ugly. *Nature Reviews Clinical Oncology*.

[20] Flanagan M. B. (2018). Primary high-risk human papillomavirus testing for cervical cancer screening in the United States: is it time?. *Archives of Pathology & Laboratory Medicine*.

[21] Musselwhite L. W., Oliveira C. M., Kwaramba T. (2016). Racial/ethnic disparities in cervical cancer screening and outcomes. *Acta Cytologica*.

[22] Rossi P. G., Baldacchini F., Ronco G. (2014). The possible effects on socio-economic inequalities of introducing HPV testing as primary test in cervical cancer screening programs. *Frontiers in Oncology*.

[23] Saslow D., Castle P. E., Cox J. T. (2007). American cancer society guideline for human papillomavirus (HPV) vaccine use to prevent cervical cancer and its precursors. *CA: A Cancer Journal for Clinicians*.

[24] Leeson S. C., Alibegashvili T., Arbyn M. (2014). HPV testing and vaccination in Europe. *Journal of Lower Genital Tract Disease*.

[25] Nanda K., McCrory D. C., Myers E. R. (2000). Accuracy of the papanicolaou test in screening for and follow-up of cervical cytologic abnormalities: a systematic review. *Annals of Internal Medicine*.

[26] Hegde D., Shetty P., Shetty H., Rai S. (2011). Diagnostic value of acetic acid comparing with conventional pap smear in the detection of colposcopic biopsy-proved CIN. *Journal of Cancer Research and Therapeutics*.

[27] Yoshida Y., Sato S., Okamura C., Nishino Y., Yajima A. (2001). Evaluating the accuracy of uterine cancer screening with the regional cancer registration system. *Acta Cytologica*.

[28] Koliopoulos G., Nyaga V. N., Santesso N. (2017). Cytology versus HPV testing for cervical cancer screening in the general population. *The Cochrane Database of Systematic Reviews*.

[29] Arbyn M., de Sanjosé S., Saraiya M. (2012). EUROGIN 2011 roadmap on prevention and treatment of HPV-related disease. *International Journal of Cancer*.

[30] Kotaniemi-Talonen L., Anttila A., Malila N. (2008). Screening with a primary human papillomavirus test does not increase detection of cervical cancer and intraepithelial neoplasia 3. *European Journal of Cancer*.

[31] Leinonen M. K., Nieminen P., Lönnberg S. (2012). Detection rates of precancerous and cancerous cervical lesions within one screening round of primary human papillomavirus DNA testing: prospective randomised trial in Finland. *BMJ*.

[32] Bulkmans N., Berkhof J., Rozendaal L. (2007). Human papillomavirus DNA testing for the detection of cervical intraepithelial neoplasia grade 3 and cancer: 5-year follow-up of a randomised controlled implementation trial. *The Lancet*.

[33] Ronco G., Segnan N., Giorgi-Rossi P. (2006). Human papillomavirus testing and liquid-based cytology: results at recruitment from the new technologies for cervical cancer randomized controlled trial. *Journal of the National Cancer Institute*.

[34] Ronco G., Giorgi-Rossi P., Carozzi F. (2006). Human papillomavirus testing and liquid-based cytology in primary screening of women younger than 35 years: results at recruitment for a randomised controlled trial. *The Lancet Oncology*.

[35] Naucler P., Ryd W., Törnberg S. (2007). Human papillomavirus and papanicolaou tests to screen for cervical cancer. *New England Journal of Medicine*.

[36] Kitchener H. C., Almonte M., Thomson C. (2009). HPV testing in combination with liquid-based cytology in primary cervical screening (ARTISTIC): a randomised controlled trial. *The Lancet Oncology*.

[37] Massad L. S. (2008). Assessing new technologies for cervical cancer screening: beyond sensitivity. *Journal of Lower Genital Tract Disease*.

[38] McCredie M. R., Sharples K. J., Paul C. (2008). Natural history of cervical neoplasia and risk of invasive cancer in women with cervical intraepithelial neoplasia 3: a retrospective cohort study. *The Lancet Oncology*.

[39] Barken S. S., Rebolj M., Andersen E. S., Lynge E. (2012). Frequency of cervical intraepithelial neoplasia treatment in a well-screened population. *International Journal of Cancer*.

[40] Lee J. W.-Y., Berkowitz Z., Saraiya M. (2011). Low-risk human papillomavirus testing and other nonrecommended human papillomavirus testing practices among U.S. health care providers. *Obstetrics & Gynecology*.

[41] Castle P. E. (2011). Abuses in human papillomavirus DNA testing. *Obstetrics & Gynecology*.

[42] Booth C. N., Bashleben C., Filomena C. A. (2013). Monitoring and ordering practices for human papillomavirus in cervical cytology: findings from the college of American Ppathologists gynecologic cytopathology quality consensus conference working group 5. *Archives of Pathology & Laboratory Medicine*.

[43] WHO Publication: HPV tests for cervical cancer screening available on https://www.paho.org/hq/index.php?option=com_content&view=article&id=11925:hpv-tests%20-for-cervical-cancer-screening%20&%20Itemid%20=%2041948%20&%20showall%20=%201%20&%20lang%20=%20en.%20Accessed%20October%202020

[44] HPV tests validated for screening. italian group for screening of cervical cancer GISCI (March 2020). Available on https://www.gisci.it/documenti/documenti_gisci/Rapporto_n5_Test_HPV_Validati.pdf

[45] Hopenhayn C., Christian A., Christian W. J. (2014). Prevalence of human papillomavirus types in invasive cervical cancers from 7 US cancer registries before vaccine introduction. *Journal of Lower Genital Tract Disease*.

[46] Giannella L., Delli Carpini G., Di Giuseppe J., Prandi S., Tsiroglou D., Ciavattini A. (2019). Age-related changes in the fraction of cervical intraepithelial neoplasia grade 3 related to HPV genotypes included in the nonavalent vaccine. *Journal of Oncology*.

[47] Blatt A. J., Kennedy R., Luff R. D., Austin R. M., Rabin D. S. (2015). Comparison of cervical cancer screening results among 256,648 women in multiple clinical practices. *Cancer Cytopathology*.

[48] McCarthy E., Ye C., Smith M., Kurtycz D. F. I. (2016). Molecular testing and cervical screening: will one test fit all?. *Journal of the American Society of Cytopathology*.

[49] Pirog E. C., Lloveras B., Lloveras B. (2014). HPV prevalence and genotypes in different histological subtypes of cervical adenocarcinoma, a worldwide analysis of 760 cases. *Modern Pathology*.

[50] Austin R. M. (2014). Can HPV primary screening reduce cervical cancer incidence and mortality?. *SCAN*.

[51] Zhou X., Sun L., Yao X., Li G., Wang Y., Lin Y. (2020). Progress in vaccination of prophylactic human papillomavirus vaccine. *Front Immunol*.

[52] WHO GLOBOCAN report 2018. Available on: https://gco.iarc.fr/today/data/factsheets/cancers/23-Cervix-uteri-fact-sheet.pdf%20accessed%20October%202018

[53] European parliamentary forum for sexual and reproductive rights. January 2020. Available on https://www.epfweb.org/sites/epfweb.org/files/hpv_and_cervical_cancer.pdf

[54] IARC 2005, Cervix cancer screening, available on https://publications.iarc.fr/Book-And-Report-Series/Iarc-Handbooks-Of-Cancer-Prevention/Cervix-Cancer-Screening-2005

[55] Chrysostomou A., Stylianou D., Constantinidou A., Kostrikis L. (2018). Cervical cancer screening programs in Europe: the transition towards HPV vaccination and population-based HPV testing. *Viruses*.

[56] Arbyn M., Anttila A., Jordan J. (2010). European guidelines for quality assurance in cervical cancer screening. second edition-summary document. *Annals of Oncology*.

[57] Arbyn M., Gultekin M., Morice P. (2020). The European response to the WHO call to eliminate cervical cancer as a public health problem. *International Journal of Cancer*.

[58] Arbyn M., Rebolj M., De Kok I. M. C. M. (2009). The challenges of organising cervical screening programmes in the 15 old member states of the European union. *European Journal of Cancer*.

[59] Italian Health System data, http://www.salute.gov.it/portale/salute/p1_5.jsp?id=27&area=Screening

[60] Vink M. A., Bogaards J. A., Meijer C. J. L. M., Berkhof J. (2015). Primary human papillomavirus DNA screening for cervical cancer prevention: can the screening interval be safely extended?. *International Journal of Cancer*.

[61] OECD Health Data 2012 Database (2011). *National Health Service *.

[62] Public Health England (2016). NHS cervical screening programme. *Colposcopy and Programme Management*.

[63] Bogaards Z., Panayotova Y., Amati C., Baili P. (2011). On behalf of the EUROCHIP working group. *Cancer Epidemiology*.

[64] Cox J. T., Castle P. E., Behrens C. M. (2013). Comparison of cervical cancer screening strategies incorporating different combinations of cytology, HPV testing, and genotyping for HPV 16/18: results from the ATHENA HPV study. *American Journal of Obstetrics and Gynecology*.

[65] Jackson B. Overtesting for cervical cancer: patterns and trends from a national reference laboratory in the United States.

[66] Huh W. K., Ault K. A., Chelmow D. (2015). Use of primary high-risk human papillomavirus testing for cervical cancer screening: interim clinical guidance. *Gynecologic Oncology*.

[67] Zhou H., Mody R., Luna E. (2014). The sensitivity of the cobas HPV Test in detecting biopsy-confirmed CIN2/3 cervical lesions: analysis of 33,857 cases with cytology and HPV co-testing. *Journal of the American Society of Cytopathology*.

[68] Workowski K. A., Bolan G. A., Centers for Disease Control and Prevention—Atlanta (2015). Sexually transmitted diseases treatment guidelines 2015. *MMWR Recommendations and Reports*.

[69] Perkins R. B., Guido R. S., Castle P. E. (2020). 2019 ASCCP risk-based management consensus guidelines for abnormal cervical cancer screening tests and cancer precursors. *Journal of Lower Genital Tract Disease*.

[70] Canadian Task Force on Preventive Health Care (2013). Recommendations on screening for cervical cancer. *CMAJ*.

[71] OECD health data 2012 database, http://www.oecd.org/health/health-systems

[72] Murillo R., Almonte M., Pereira A. (2008). Cervical cancer screening programs in Latin America and the Caribbean. *Vaccine*.

[73] Villa L. L. (2012). Cervical cancer in Latin America and the Caribbean: the problem and the way to solutions. *Cancer Epidemiology Biomarkers & Prevention*.

[74] Hammond I., Saville M. (2020). Cancer council Australia cervical cancer screening guidelines working party. *National Cervical Screening Program: Guidelines for the Management of Screen-Detected Abnormalities, Screening in Specific Populations and Investigation of Abnormal Vaginal Bleeding *.

[75] Hamashima C., Aoki D., Miyagi E. (2010). The Japanese guideline for cervical cancer screening. *Japanese Journal of Clinical Oncology*.

[76] Kobayashi D., Takahashi O., Hikosaka C., Okubo T., Fukui T. (2013). Optimal cervical cytology mass screening interval for cervical cancer. *Archives of Gynecology and Obstetrics*.

[77] Palcic C. (2011). Cervical screening in China: project management considerations. *International Cost Engineering Council (ICEC)*.

[78] Black E., Richmond R. (2018). Prevention of cervical cancer in sub-saharan Africa: the advantages and challenges of HPV vaccination. *Vaccines*.

[79] Msyamboza K. P., Phiri T., Sichali W., Kwenda W., Kachale F. (2016). Cervical cancer screening uptake and challenges in Malawi from 2011 to 2015: retrospective cohort study. *BMC Public Health*.

[80] Beddoe A. M. (2019). Elimination of cervical cancer: challenges for developing countries. *Ecancermedicalscience*.

[81] Cousins S. (2019). https://mosaicscience.com/story/rwandacervical-cancer-hpv-vaccine-gardasil-cervarix/.

[82] Sancho-Garnier H., Khazraji Y. C., Cherif M. H. (2013). Overview of cervical cancer screening practices in the extended Middle East and North Africa countries. *Vaccine*.

[83] Osgul R., Mbele M., Makhafola T. (2020). Cervical cancer in low and middle‑income countries (Review). *Oncology Letters*.

[84] Mehrotra N., Naidoo M., Moodley I. (2015). Human papillomavirus (HPV) vaccination of adolescents in the South African private health sector: lessons from the HPV demonstration project in KwaZulu-Natal. *South African Medical Journal*.

[85] Jayant K., Sankaranarayanan R., Thorat R. V. (2016). Improved survival of cervical cancer patients in a screened population in rural India. *Asian Pacific Journal of Cancer Prevention: APJCP*.

[86] Ali S., Skirton H., Clark M. T., Donaldson C. (2017). Integrative review of cervical cancer screening in western asian and middle eastern Arab countries. *Nursing & Health Sciences*.

[87] Cheung L. C., Egemen D., Chen X. (2020). 2019 ASCCP risk-based management consensus guidelines: methods for risk estimation, recommended management, and validation. *Journal of Lower Genital Tract Disease*.

[88] Egemen D., Cheung L. C., Chen X. (2020). Risk estimates supporting the 2019 ASCCP risk-based management consensus guidelines. *Journal of Lower Genital Tract Disease*.

[89] Perkins R. B., Fuzzell L. N., Lake P. (2020). Incorporating stakeholder feedback in guidelines development for the management of abnormal cervical cancer screening tests. *Journal of Lower Genital Tract Disease*.

[90] Bonde J. H., Sandri M.-T., Gary D. S., Andrews J. C. (2020). Clinical utility of human papillomavirus genotyping in cervical cancer screening: a systematic review. *Journal of Lower Genital Tract Disease*.

[91] Farag S., Fields J., Pereira E., Chyjek K., Chen K. T. (2016). Identification and rating of gynecologic oncology applications using the applications scoring system. *Telemedicine and e-Health*.

[92] Moodley J., Constant D., Botha M. H., van der Merwe F. H., Edwards A., Momberg M. (2019). Exploring the feasibility of using mobile phones to improve the management of clients with cervical cancer precursor lesions. *BMC Women’s Health*.

